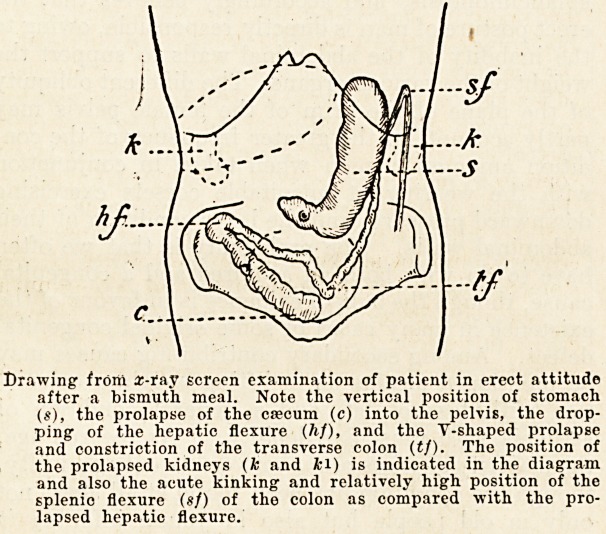# Splanchnoptosis (Enteroptosis)

**Published:** 1911-09-16

**Authors:** R. Murray Leslie

**Affiliations:** Physician Prince of Wales's General Hospital.


### HOSPITAL CLINICS.

### SPLANCHNOPTOSIS (ENTEROPTOSIS).

By R. MURRAY LESLIE, M.A., B.Sc., M.D., M.R.C.P., Physician Prince of Wales's General Hospital.

(Lecture delivered at the North-East London Post-Graduate College.)

It is a remarkable fact that dropping or downward displacement of the abdominal organs, which is now universally recognised as one of the commonest clinical conditions met with in general practice, has until quite recent years been almost unknown as a clinical entity.

I even remember well as a student hearing the great anatomist, Sir William Turner, state that in his large experience he had rarely come across a case of so-called movable kidney. As far back, however, as 1853, the great pathologist, Virchow, described displacement of the intestines, which he at that time ascribed to local peritonitis and which appeared to be the cause of certain forms of dyspepsia. It was not, however, until 1885 that Dr. Glenard, of Vichy, gave the first complete description of enteroptosis and which, therefore, often goes by the name of "Glenard's Disease." This observer reported no less than 148 cases amongst 1,300 persons undergoing the cure at Vichy under his direction, and in most of these he associated the condition with a certain type of nervous dyspepsia.

Recent writers prefer the term splanchnoptosis as the generic title, and use the terms gastroptosis, nephroptosis, enteroptosis, hepatoptosis, or splenoptosis according as to whether the displacement, entirely or mainly, affects the stomach, kidney, bowel, liver, or spleen.

### INCIDENCE.

Splanchnoptosis is much more frequently met with in women. It is said to be ten times more frequent in females. Einhorn found the condition in no less than 33.25 per cent. of his women patients, an observation which has been confirmed by other observers. If these observations are correct, one out of every three women suffers from some degree of displacement of one or more organs, more particularly the right kidney and intestine. In the great majority of cases dropping of one organ is associated with more or less displacement of other organs.

As regards causation, many authorities believe that the condition is frequently congenital. Thus Glenard himself thought that the main cause is a constitutional weakness or looseness of the mesenteric and peritoneal attachments; while Landau, on the other hand, thought that the primary cause is a congenital weakness of the abdominal wall. A particular body type has been described—a slender skeleton, a long thorax, soft, flabby abdominal muscles, a defective development of the panniculus adiposus, and very frequently a floating tenth rib. Dr. Harris goes so far as to give actual figures, and states that if you divide the distance from the supra sternal notch to the symphysis pubis by the least circumference of the abdomen and multiply by 100 you get an average index of 77; and that if the index is above 77, there is generally nephroptosis or some other form of visceral displacement. According to this observer, therefore, the main congenital abnormality is a marked contraction of the base of the thorax and upper part of abdomen.

### ACQUIRED DISPLACEMENT.

Other observers, however, are equally emphatic in stating that splanchnoptosis is often an acquired condition. Thus Einhorn, who has made a special study of the subject, lays stress upon the corset as being one of the important causal factors, particularly the corset which exerts pressure on the base of the thorax and upper portion of the abdomen. In another important group of cases the condition is the result of relaxation of the abdominal muscles and stretching of the hepatocolic and other suspending ligaments following repeated pregnancies. Keith believes that the condition is due to wrong methods of respiration. Another observer explains the abnormality from the standpoint of evolution; he has found that quadrupeds rarely suffer from splanchnoptosis, and accordingly believes that the erect posture of man is directly responsible, owing to the inability of the abdominal walls to support the weight of the various organs. The different obliquity of the plane of the brim of the female pelvis may partly account for the greater frequency of the condition amongst women when taken in conjunction with the wearing of unsuitable corsets exercising downward pressure, and the laxer condition of their abdominal walls. The probability is that we often have to do with both an acquired and a congenital cause, though the bulk of evidence is in favour of the existence in many cases of some original congenital defect. Among secondary contributing causes may be mentioned strains as in lifting weights, emaciation with loss of fat, and a general condition of asthenia with muscular debility. As regards age, the majority of cases occur between twenty and fifty, though the condition has been found at all ages, not only in old people but also in young infants: it has even been known in quite young infants, which is a strong argument in favour of its frequent congenital origin.

### SYMPTOMS.

The symptoms of splanchnoptosis may be divided into two groups—(1) *local symptoms* connected with the displaced organ or organs, and (2) *symptoms of neurasthenia*. Before considering these groups, it is well to state that both local and general symptoms are, as a rule, in no sense proportional to the actual visceral displacement. Thus the condition may be extreme with practically no symptoms whatever, or the patient may have a few vague nervous symptoms indicating a slight degree of neurasthenia, which induce her to consult a physician, who then discovers the abnormal position of the organs quite accidentally in the course of his routine examination. In such a case it is often unwise to draw the patient's attention to the abnormality unless a remark is added that many women have this condition without it causing the least inconvenience. If too much is made of the matter in these mild cases, there is a risk of some of these persons relapsing into chronic invalidism with obstinate neurasthenia, which may be the cause of great misery to the patients themselves and to their domestic circles.

When well marked symptoms are present, there is often pain or discomfort in one or other region of the abdomen, accompanied by a varying degree of neurasthenia. The patient is often a woman, somewhat pallid, emaciated, and with a curious nervous and juvenile expression. She has usually lax, flabby abdominal muscles with marked flattening of the abdomen, with a general appearance of constriction in the lower part of the chest, and occasionally a floating tenth rib. There is often pain or discomfort which may take the form of backache, sideache, gastralgia, pain on passing water, and occasionally nausea and vomiting. These symptoms are usually aggravated by movement or exertion, and frequently the patient is unable to stand or walk for any length of time without considerable discomfort and consequent fatigue. The symptoms may entirely disappear on lying down, and are nearly always diminished by raising the abdomen by means of some support. Glenard's test was to stand behind

Drawing from *x*-ray screen examination of patient in erect attitude after a bismuth meal. Note the vertical position of stomach (*s*), the prolapse of the cæcum (*c*) into the pelvis, the dropping of the hepatic flexure (*hf*), and the V-shaped prolapse and constriction of the transverse colon (*tf*). The position of the prolapsed kidneys (*k* and *k*1) is indicated in the diagram and also the acute kinking and relatively high position of the splenic flexure (*sf*) of the colon as compared with the prolapsed hepatic flexure.

the patient and with both hands to lift up the abdomen from below, when the pain and discomfort were often instantly relieved. Glenard laid great stress upon the cord-like thickening representing the contracted band of the transverse colon, through which the aortic pulsation can generally be distinctly felt. He also noticed the frequency of movable kidney on the right side and splashing sounds in the prolapsed stomach. Glenard believed that the starting point was the dropping of the hepatic flexure of the colon due to relaxation of the hepato-colic ligament. A special symptom noticed by some observers is the frequency of pain and fatigue during the hours of duodenal digestion. Amongst the neurasthenic symptoms, so commonly associated with splanchnoptosis, may be mentioned general malaise, inertia and incapacity for work, irritability, lack of power of concentration, depression of spirits, loss of memory, sleeplessness and various hysterical manifestations.

The above diagram illustrates a very typical case now under my care. The patient is a thin, unmarried woman of forty years of age, who first consulted me some two years ago for symptoms of neurasthenia, with a history of having recently undergone a rest cure in a nursing home for this condition. Twelve years previously she had been under the care of Sir Lauder Brunton, who then diagnosed movable kidney. She was also seen by Sir Frederick Treves, who did not consider it necessary to operate, and she was accordingly treated by a special kidney belt. She remained fairly comfortable for three years, when she had a complete nervous collapse due to an emotional shock. This was nine years before she came under my observation. She remained delicate and complained of nervous debility and occasional severe pain in the back and right loin. I found her suffering from numerous neurasthenic symptoms—languor and feeling of exhaustion, general inertia, irritability and one or two 'phobias, notably a dread of crowded places. The skin had an unhealthy pallor; there were both pain and tenderness in the right loin, and a certain amount of flatulent dyspepsia. The diagram illustrates the position of the abdominal viscera. The kidneys are sketched in to indicate their position as discovered by palpation. It will be seen that the right kidney is much prolapsed, while the left was readily palpable well below the costal margin, and both were freely movable. The position of the stomach and bowel was determined by *x*-ray examination, after the administration of bismuth. The stomach is shown as lying almost vertical, with the pyloric extremity lying underneath the umbilicus. The cæcum is right down in the true pelvis, while the hepatic flexure of the colon lies below the level of the iliac crest. The transverse colon was somewhat constricted and prolapsed in a V-shaped fashion, the apex of the V being also down in the pelvis. The splenic flexure of the colon was practically in its normal position, situated close to the cardiac end of the stomach, where it formed an acute angle with the descending colon, being maintained in position by the splenico-colic ligament. It may be said in passing that it is not commonly understood how high up the splenic flexure of the colon normally is. The *x*-ray examination was made with the patient in the erect attitude. The special body type already alluded to with the constricted lower part of the chest and the flattened abdomen was particularly characteristic.

At this stage it may be well to briefly describe the symptoms specially characteristic of displacement of particular abdominal organs. In *gastroptosis* (prolapse of the stomach) the organ, as in the case just described, frequently occupies a semi-vertical position, the pyloric end descending while the cardiac end is fixed. There may be no symptoms or there may be the ordinary symptoms of dilatation and delayed digestion, namely, flatulence and distention after meals, loss of appetite, coated tongue, nausea and sometimes vomiting, occasional gastralgia, giddiness and tachycardia and commonly a diminution of hydrochloric acid; splashing can generally be elicited on palpation of the stomach. It is found that dilatation is very likely to occur when the stomach assumes the vertical form just described. There may be well marked associated kinking at the pyloro-duodenal junction, which may partly account for the gastric dilatation.

### ENTEROPTOSIS.

As regards *enteroptosis*, it may be stated that a certain degree of displacement of intestines is found in all cases of splanchnoptosis. The colon is generally prolapsed (coloptosis), the transverse colon being frequently bent in a U- or V-shaped fashion. As a result there is kinking of the hepatic and splenic flexures, particularly the splenic, where the angle is often extremely acute. Kinks are also common at the duodeno-jejunal and ileocæcal junctions. The frequency of constipation in women is believed by Ewald to be largely due to the kinks produced in connection with enteroptosis. He is of opinion that in addition to the actual mechanical obstruction these kinks and the dragging of peritoneal bands produce reflex irritation of the bowel and consequent interference with its motor and secretory functions. Mr. Arbuthnot Lane believes that in connection with this constipation there are formed numerous adhesions which produce a condition of chronic intestinal stasis with its accompanying phenomena of auto-intoxication from intestinal absorption. It is to this auto-intoxication that he attributes the loss of fat, the cardiac degeneration and consequent impaired circulation manifested by cold, clammy, and often livid hands and feet, the frequent dark staining of the skin, particularly in certain regions, the muscular debility, the dull headache and incapacity for work, and the mental depression often amounting to mild melancholia. Mr. Lane is further of opinion that the formation of abnormal mesenteric and other peritoneal adhesions leads to all sorts of other undesirable complications, including kinking of the appendix with appendicitis, disturbance of intestinal secretion leading to mucous colitis, constrictions of the ovary leading to subsequent cystic disease, and mechanical interference with the function of the bladder and gall duct facilitating the formation of gall stones. Keith, on the other hand, suggests that the frequency of gall stones in cases of splanchnoptosis is due to slight prolapse of the liver producing a descent of the duodenum, head of the pancreas, and termination of the common bile duct, which thus make the entrance and exit of the bile to and from the gall bladder extremely difficult. Backward displacements of the uterus and prolapse of the ovary are frequently met with. According to one observer, 80 per cent. of women with enteroptosis have uterine retroversion. Pain and fatigue during the hours of duodenal digestion are characteristic symptoms. The actual diagnosis of enteroptosis is best made by an *x*-ray examination of the bowel after the previous administration of a bismuth meal.

### KIDNEY PROLAPSE.

*Nephroptosis* (prolapse of the kidney) is by far the commonest form of splanchnoptosis, and is certainly the one which has attracted most attention. There are often no symptoms whatsoever, as will be readily understood when it is remembered that one in every three women have a displaced kidney which is often discovered quite accidentally. There are all degrees of mobility of the kidney; the organ may be just palpable below the costal margin, or it may be freely moveable in every direction, and may be actually found in the pelvis. The bimanual examination is the only satisfactory method of diagnosis, and the patient should be examined lying on her back and on either side and also in the standing position. As regards symptoms, they are in no sense proportional to the degree of displacement. It is stated that only one out of ten cases of moveable kidney has distinct local symptoms; when these are present they are usually found on the right side, the marked preponderance of right-sided displacements being usually attributed to the position of the right kidney just below the heavy liver, through the medium of which it is subjected to constant movements of depression with each descent of the diaphragm during inspiration. The following are the principal symptoms associated with nephroptosis—one or more of which may be present in any individual case. There may be merely a feeling of lack of support in the loin or back, or a dragging dull pain, continuous or intermittent, in the same region, often relieved by lying down. There may be frequency of micturition, sometimes associated with a burning sensation in the vulva. In a certain number of cases we have the occasional recurrence of the so-called Dietl's crises, consisting of paroxysmal attacks of intense pain, nausea, and vomiting, supposed to be the result of kinking of the ureter or torsion of the renal vessels and nerves; during the attack the urine may become high-coloured and loaded with urates, and there is occasionally intermittent hæmaturia and albuminuria. In not a few cases, after the lapse of some years, a condition of hydronephrosis may be developed, characterised by the usual disappearing swelling with intermittent polyuria. In some instances chronic appendicitis is found associated with displaced right kidney, and it is often advisable in certain cases to remove the appendix at the same time as the kidney is stitched up in position. A floating kidney is sometimes associated with obstruction of the common bile duct. In even mild cases reflex nausea and vomiting may occur in association with the aching pain in the right loin or back. The displaced kidney is often very tender on palpation.

### LIVER AND SPLEEN.

*Hepatotosis* and *splenoptosis* are much less common. I recently saw a well-marked case of the former condition where the lower edge of the liver was found at the level of the umbilicus, while percussion revealed the upper border as close to the right costal margin. The condition in some cases is undoubtedly to be attributed to tight lacing. The condition is clinically important as it is generally mistaken for an enlarged liver. There may be no symptoms, or on the other hand there may occasionally be pain or discomfort in the right hypochondrium or right shoulder with flatulence or other dyspeptic symptoms. There is sometimes a bearingdown sensation below the right costal margin and occasionally symptoms suggesting gall stones. An examination of the patient in the erect attitude reveals a bulging below the right costal margin due to the convex upper surface of the organ. The spleen may be very much displaced and is usually associated with other forms of splanchnoptosis. It is sometimes so low down that it has on more than one occasion been mistaken for a tumour of one of the pelvic organs.

### Prognosis.

The prognosis of splanchnoptosis varies greatly with individual cases. In some instances the symptoms may entirely disappear; while in other cases all forms of therapeutic measures may fail to give permanent relief. In the majority of instances, however, the symptoms may generally be greatly alleviated and often removed by appropriate treatment.

The importance of prophylaxis or preventive treatment must not be forgotten. Great care should be exercised in not permitting women with the characteristic body form already described to get up too soon after confinements or after any debilitating illness. If the patient is thin with relaxed abdominal muscles she should rest in the recumbent attitude for one or two hours daily, and should on no consideration wear corsets that exert pressure upon the upper part of the abdomen. She should be specially cautioned against lifting heavy weights such as large pieces of furniture. Much permanent damage may be done in the course of a vigorous but injudicious "spring-cleaning." One patient came under my observation where dislocation of the right kidney was produced by an attempt to open the lower sash of a window which was stiffened and fixed in position through long disuse. If there is a tendency to emaciation milk and other fattening foods ought to be partaken of freely so as to produce the requisite amount of adipose padding.

### Treatment.

As regards treatment when the condition is once established, I strongly believe in the great value of abdominal massage and exercises for the abdominal muscles in conjunction with rest in the prone or supine position during some hours of each day. I have found that in quite a number of cases the strengthening of the abdominal walls is all that is necessary to secure the adequate support of the abdominal organs. The massage should be administered by a trained masseuse, and I have found electrical stimulation to be an important help in the same direction. The abdominal exercises should be performed, both in the lying and in the standing position. An excellent exercise is to clasp the hands together behind the neck in the supine position, raise the extended legs as high as possible and slowly let them fall and then raise them again just before they reach the ground; or vice versa keep the legs extended on the ground and slowly sit up with the arms stretched forward until the fingers touch the feet and then slowly lie down again. In the standing posture extend the arms above the head, and with the legs rigidly extended bend the trunk forward and try to touch the toes with the fingers. Other movements in the erect attitude include bending first to one side and then to the other, twisting the body round first in one direction and then in the other and leaning back; while a particularly valuable exercise is to slowly assume the squatting position, standing on the toes with the knees bent outwards and then slowly rising again.

### Supports.

The most fashionable, and in some cases the most efficacious treatment, consists in the wearing of specially designed mechanical supports, either in the form of belts or corsets. The great principle is to increase intra-abdominal pressure, taking care that the pressure of the bandage, belt, or corset shall be exerted over the lower part of the abdomen, the direction of pressure being backwards and upwards. Mr. Ernst, of Charlotte Street, has designed an excellent enteroptosis belt or appliance which is intended to exert the same pressure as would be exerted by Glenard's two hands applied while standing behind the patient. This is, however, a somewhat expensive appliance, and quite good results may often be obtained by a well-adapted linen abdominal bandage with straps so arranged as to prevent the bandage rising up over the hips.

### Operation.

As regards the operative treatment for splanchnoptosis, surgical measures are only warranted after hygienic, dietetic and mechanical measures have been given a fair trial. In regard to nephroptosis, the indications for operation are more or less constant pain producing chronic invalidism, Dietl's crisis and intermittent hydronephrosis. The mortality of nephrorrhaphy is practically nil; indeed, Sir Henry Morris had no deaths in nearly two hundred consecutive cases. In advanced enteroptosis with intestinal kinks and obstinate constipation due to the resulting intestinal stasis the operation of short-circuiting of the ileum into the sigmoid flexure or rectum and in aggravated cases the resecting the intervening portion of the prolapsed colon may be performed with great advantage. In the case of one of my patients Mr. Lane resected the cæcum, and also the ascending, transverse, and descending colons after short-circuiting the ileum into the lower portion of the sigmoid. The patient—an unmarried woman thirty years of age—from being a hopeless chronic invalid now enjoys almost perfect health, and is able to play seven consecutive sets of tennis without undue fatigue. Similar plastic operations have been performed in the case of the stomach, liver, and spleen with considerable success.

In conclusion, the physician must not forget the frequently associated neurasthenia which, if pronounced, may have to be dealt with by modified Weir Mitchell or other appropriate treatment. At the same time, as a matter of experience, it is frequently found that the relief of the local symptoms will largely contribute to the disappearance of the various neurasthenic manifestations.

## Figures and Tables

**Figure f1:**